# Paragonimiasis Misdiagnosed as Pulmonary Tuberculosis: A Case Report

**DOI:** 10.7759/cureus.36169

**Published:** 2023-03-15

**Authors:** Renzo Villanueva-Villegas, Javier Diaz-Mendoza, Juan Salas-Lopez, Carlos Espiche

**Affiliations:** 1 Pulmonary Medicine, Hospital Nacional Dos de Mayo, Lima, PER; 2 Institute of Research in Biomedical Sciences, Faculty of Medicine, Ricardo Palma University, Lima, PER; 3 Pulmonary and Critical Care Medicine, Henry Ford Hospital-Wayne State University, Detroit, USA; 4 Faculty of Medicine, Universidad Nacional Mayor de San Marcos, Lima, PER; 5 Medicine, SBH Health System/CUNY School of Medicine, Bronx, USA

**Keywords:** pulmonary disease, parasit, zoonosis and public health, pulmonary tuberculosis, lung fluke

## Abstract

Paragonimiasis is a zoonosis caused by the ingestion of raw or undercooked crustaceans parasitized with metacercaria of *Paragonimus spp*. In Peru, Cajamarca is considered an endemic region for paragonimiasis. A 29-year-old man from the department of San Martín, Peru presented with a three-year history of cough, chest pain, fever, and hemoptysis. Treatment for tuberculosis (TB) was initiated even with negative results for sputum acid-fast bacillus (AFB), due to the patient's clinical characteristics and the high prevalence of the condition in the region. After eight months, due to the lack of any clinical improvement, he was referred to a regional hospital, where *Paragonimus* eggs were evidenced in direct sputum cytology. The patient received treatment with triclabendazole and showed clinical and radiological improvement. It is important to consider eating habits, even in non-endemic areas, for diagnosing paragonimiasis in patients with TB symptoms who do not respond to a specific treatment.

## Introduction

*Paragonimus* is prevalent in the Far East, West Africa, and the Americas, including Peru, and infest millions worldwide. This trematode or fluke causes paragonimiasis and is more frequently found in areas where intermediate hosts such as snails, crabs, and crayfish live and where undercooked or raw seafood is served [1]. The most common species that infect humans is *Paragonimus westermani*, but many other species have also been reported to affect humans [2].

The most common presentation of paragonimiasis includes extrapulmonary and pulmonary symptoms; the most frequent ones include cough, chest pain, and fever. The infection could sometimes affect the central nervous system and cause more severe symptoms. Foodborne trematode infections cause a high rate of disability worldwide every year [3].

The differential diagnosis in an individual with cough and hemoptysis includes infectious and non-infectious diseases. Pulmonary tuberculosis (TB) is also on the list of differential diagnoses due to its high prevalence in Peru; it manifests with recurrent hemoptysis and cavitation, predominantly apical lesions. TB is an infection caused by the bacteria *Mycobacterium tuberculosis*. This infection is prevalent in Peru, and it is one of the most affected countries in Latin America [4]. While TB can affect several organs, it mainly affects the lungs. The majority of infections are asymptomatic and stay as latent TB. If TB is active, it commonly presents as cough, fever, night sweats, and weight loss [5]. TB affects approximately 33,000 people in Peru and kills 4,000 annually [6].

The correct diagnosis and treatment can help achieve favorable outcomes in these patients. Recognizing the condition based on epidemiological findings could be challenging, and this case report aims to illustrate the various aspects of the diagnostic and treatment process.

## Case presentation

**Figure 1:**
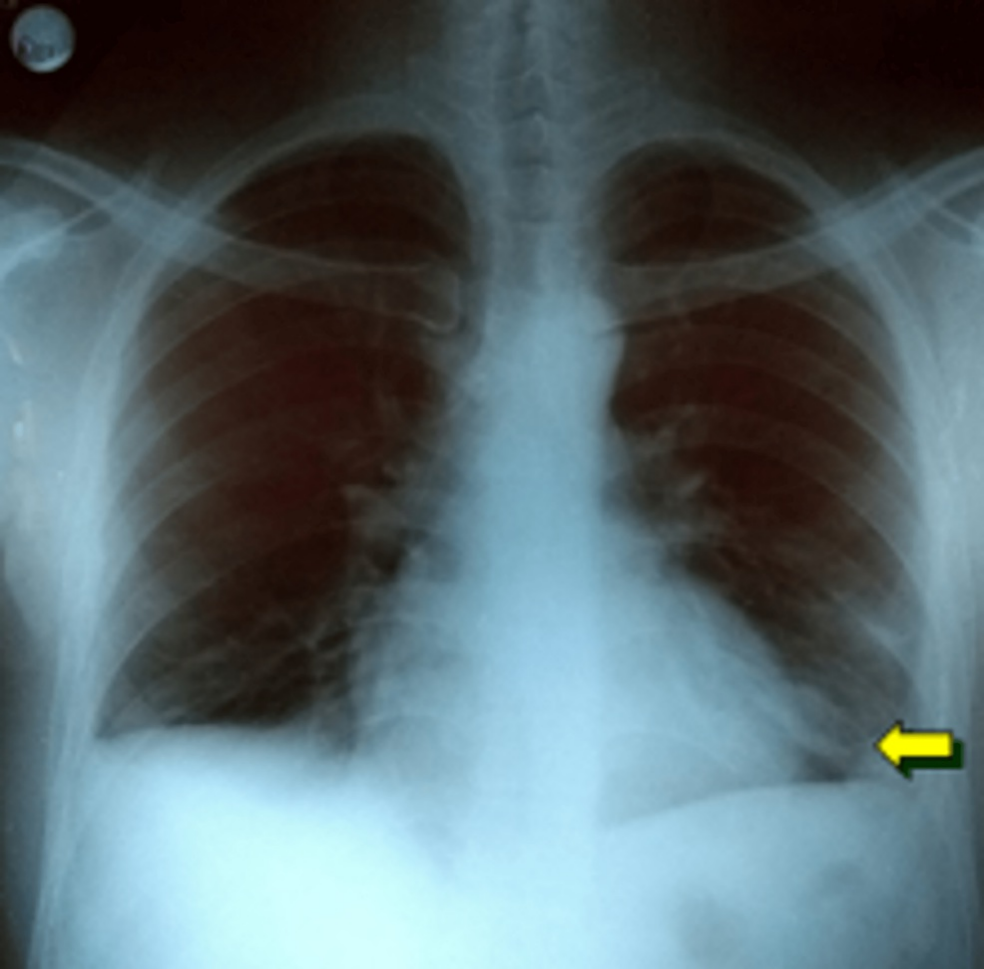
Chest radiograph revealed a heterogeneous, cavitated lesion in the left hemithorax (positive radiologic Felson sign)

A 29-year-old man from the Peruvian Amazon with no known medical history presented with three years of progressive cough, thoracic pain, fever, and hemoptysis. The chest radiograph revealed a heterogeneous, cavitated nodule in the left hemithorax, obliterating the left heart border (Figure 1).

Despite a negative acid-fast bacillus (AFB) in sputum, the patient received empiric treatment for pulmonary TB for eight months, given its high prevalence in Peru. Due to a lack of radiological and clinical response, the patient was referred to our hospital for further management. The physical exam was significant for diminished vesicular murmur on the lower left hemithorax. Laboratory studies revealed a white blood cell count of 8930/μl with eosinophils of 1198/μl (13%). The autoimmune panel showed normal C-reactive protein (CRP) at 6.9 mg/L (normal range: 0-10 mg/L), erythrocyte sedimentation rate (ESR) of 5 mm/h (normal range: 0-22 mm/h), rheumatoid factor of 8 UI/ml, as well as negative antinuclear antibodies (ANA) and antineutrophil cytoplasmic autoantibody, cytoplasmic (c-ANCA).

**Figure 2:**
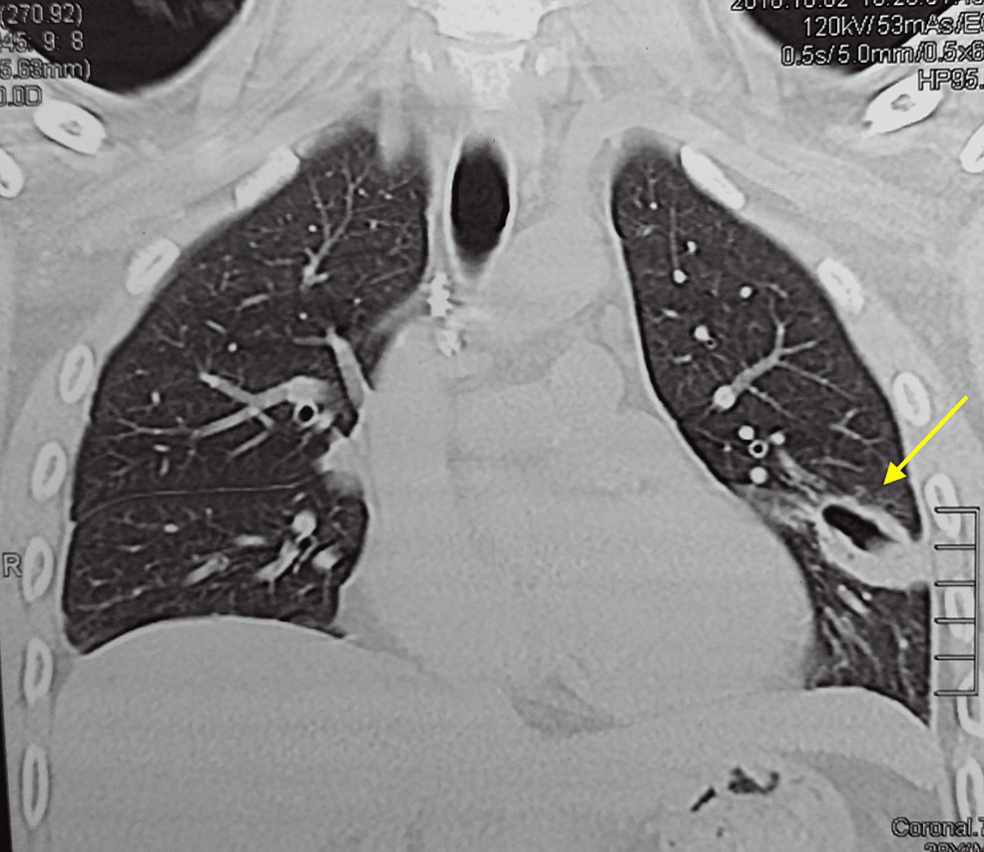
CT images revealed a 2.6 x 3-cm lingular cavitated lesion with thick walls, as well as fibrous tracts towards the visceral pleura CT: computed tomography

Additionally, an exhaustive workup was done, and the complete blood count (CBC) revealed hypereosinophilia and polycythemia while the complete metabolic panel (CMP) was within normal limits. The tests for HIV, rapid plasma reagin (RPR), and rheumatoid factor were negative. GeneXpert was negative for TB; bronchoscopy with biopsy was negative for TB or neoplasm, and bronchoalveolar lavage (BAL) was also negative. Other findings included negative fungi and common germs sputum culture, and negative hepatitis C and B. CT images revealed a 2.6 x 3-cm lingular cavitated lesion with thick walls and fibrous tracts towards the visceral pleura (Figure 2). An inquiry into the patient's dietary habits revealed that he regularly consumed raw crabs and shrimp.

Pulmonary TB was ruled out due to the negative test described above, the lack of response to empirical treatment (rifampicin, pyrazinamide, ethambutol, and isoniazid), and the presence of eosinophilia indicating a parasitic infection. Aspergillosis often presents with fever, pleuritic chest pain, and hemoptysis. It typically occurs in immunosuppressed individuals.

**Figure 3:**
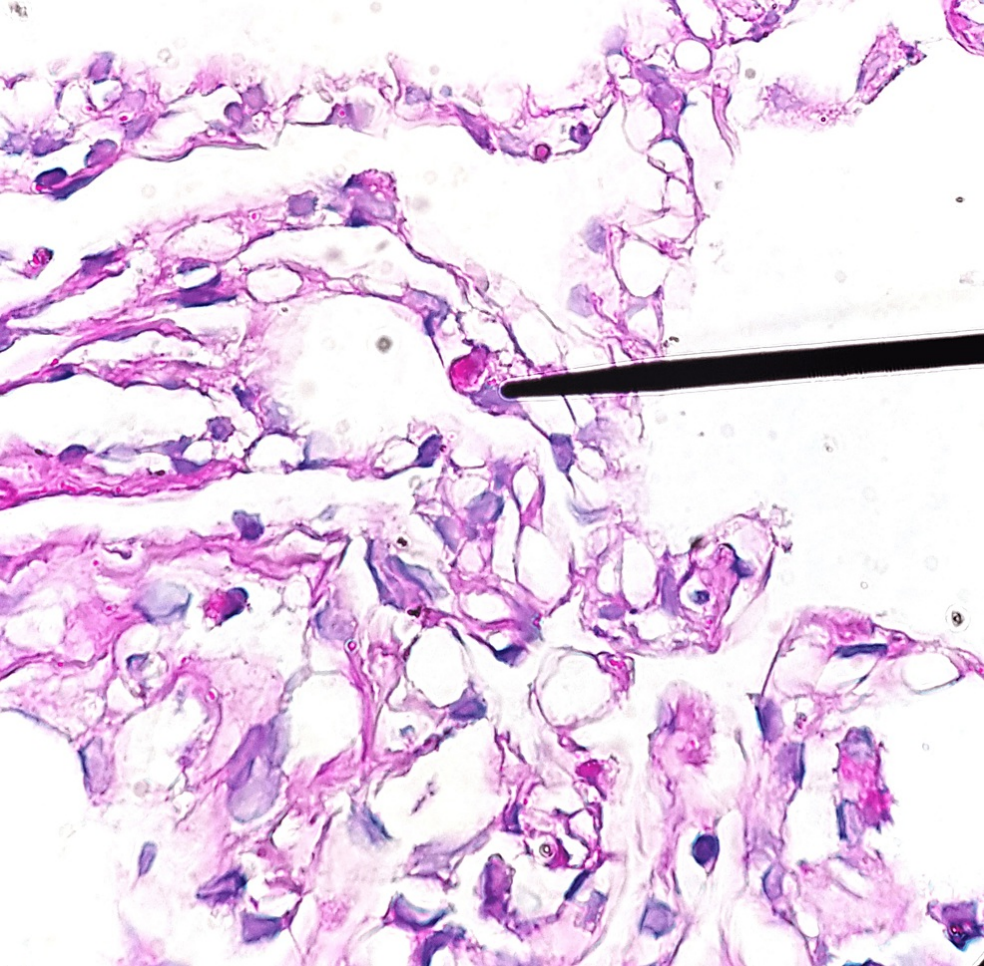
Transbronchial lung biopsies of the affected area revealed the presence of eosinophils

**Figure 4:**
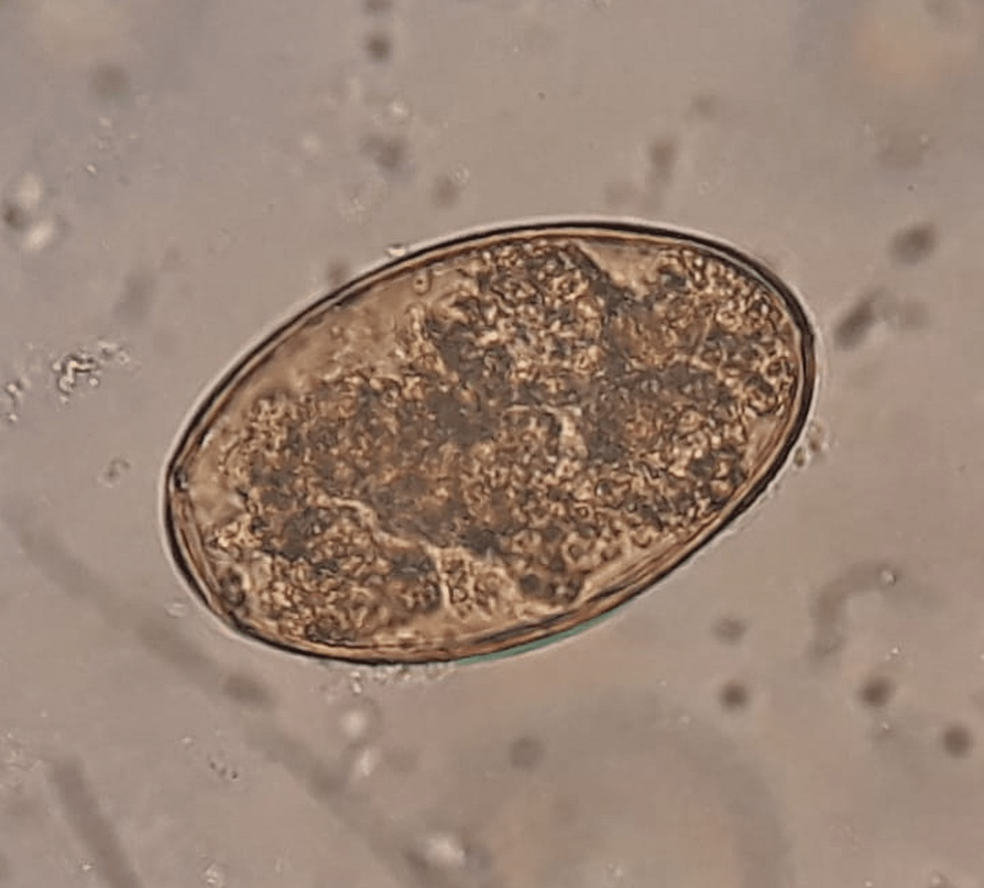
Eggs of Paragonimus spp.

**Figure 5:**
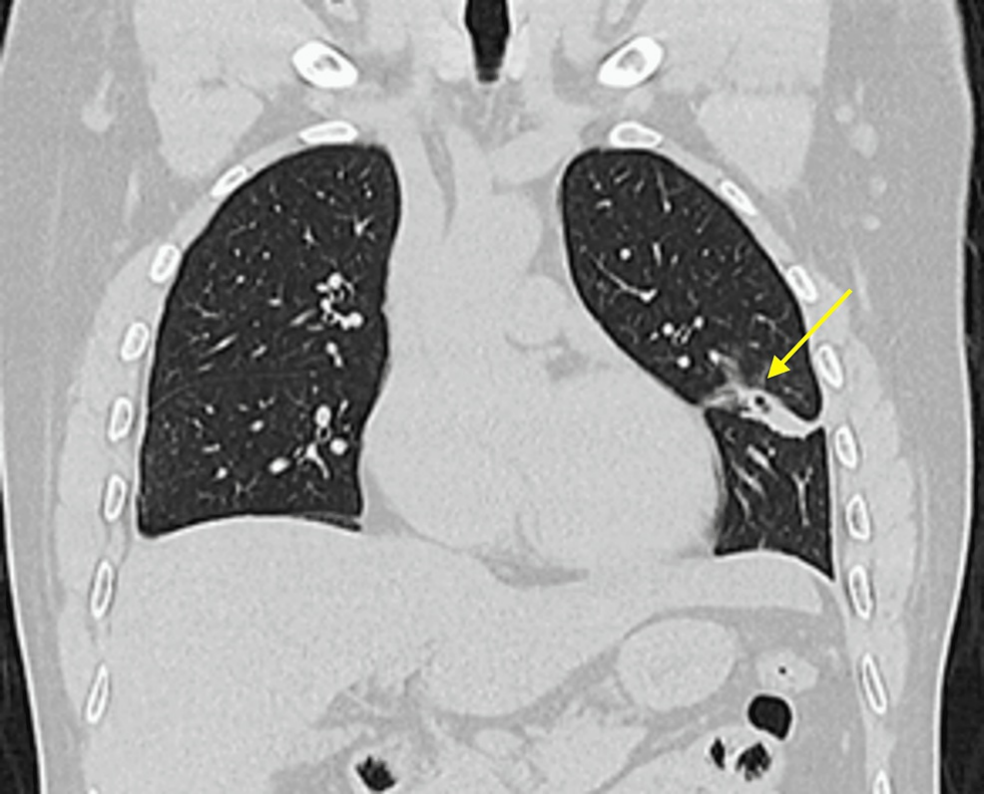
Repeat lung CT showed a decrease in the size of the cavitated subpleural lesion at seven days of treatment CT: computed tomography

Transbronchial lung biopsies of the affected area were negative for TB and neoplasm but revealed the presence of eosinophils in the tissue (Figure 3). AFB in sputum was negative. Bacterial and fungal cultures were unremarkable. Based on the patient's dietary habits which included the consumption of raw crabs and shrimps, as well as the presence of eosinophilia, a high suspicion for paragonimiasis was maintained. Sputum cytology showed *Paragonimus spp. *eggs (Figure 4). The patient received treatment with triclabendazole 1250 mg in a single dose with significant clinical improvement. Additionally, a repeat lung CT showed a decrease in the size of the cavitated subpleural lesion (Figure 5). The patient was subsequently discharged after seven days of treatment.

## Discussion

Paragonimiasis is a parasitic disease caused by a trematode of the genus *Paragonimus, *and the World Health Organization (WHO) has described it as one of the neglected tropical diseases worldwide [7]. In 2005, this parasitic disease affected more than 22 million people in 48 countries worldwide, mainly in Asia; and as many as 173 and 630 cases were reported in Peru and Ecuador, respectively [8].

In Peru, Cajamarca in the northeast region of the country is recognized as an endemic area for ​​paragonimiasis. Cases have also been reported in the departments of La Libertad and Lima [9,10]. However, no case of paragonimiasis had been previously reported in the Peruvian Amazon; one of the hypotheses pertains to the presence of immigrants from endemic areas of the disease. According to a report on internal migrations in Peru at the departmental level in 2015 by the National Institute of Statistics and Informatics (INEI), the immigrant population to the department of San Martín (where our index patient comes from) comes mainly from the department of Cajamarca, accounting for approximately 45.6% of all the internal immigrant population [11].

Paragonimiasis in humans results from the consumption of raw freshwater crab or crayfish infected with metacercaria. In the case of *P. mexicanus* and *P. peruvianus*, they cross the duodenum wall and then move to the intercostal and/or abdominal muscles. After a short period, they migrate to the lung parenchyma where they form cavities [12,13]. The adult worm is usually located in the lung parenchyma. The cavity that is formed there communicates with the bronchi and thus reaches the upper airways and is eliminated in the sputum. In some cases, they pass into the digestive tract and are eliminated in the stool. In most species, the parasite is oval-shaped at this stage, similar to a coffee bean, 8-15 mm long and 4-5 mm wide [14].

In the initial phase of this disease (8-10 weeks), after the parasite has crossed the diaphragm and affected the pleura, eosinophilic exudate, pneumothorax, and pleuritic pain become evident. When the invasion affects the lungs, cough, low fever, hemoptysis, and migratory infiltrate occur. The late phase, which can last from 6 to 20 years, is characterized by encystment of the parasite in the lung parenchyma, which is radiographically evidenced as bronchiectasis or fibrous tracts. During this phase, the parasite produces about 20,000 eggs daily and is clinically characterized by persistent cough with chocolate-brown expectoration [15].

The diagnostic approach depends on the stage of infection, the nature of clinical manifestations, and the diagnostic tools available. Nonspecific findings may include peripheral eosinophilia and a raised IgE. Establishing the diagnosis of paragonimiasis during early infection (before egg production) is difficult. It can be made only presumptively based on compatible clinical manifestations in a patient with eosinophilia and pertinent exposure history. During late infection, the diagnosis is suggested by a history of recurrent hemoptysis in a patient with epidemiologic risk factors. It can usually be confirmed by identifying *Paragonimus* eggs in the sputum or BAL in the late phase of infection. Since these clinical manifestations are similar to those of TB or lung malignancy, many paragonimiasis patients are initially treated for other diseases due to misdiagnosis [14]. The unique aspect of our case is that the patient comes from a non-endemic area for paragonimiasis. Initially, the main differential diagnosis was TB based on its high prevalence in Peru.

Radiological features include the combination of pleural effusion (present in 20-60% of cases) and multiple cysts, irregular linear lesions, or nodular opacities in the lung parenchyma [15]. In previous reports, pleural lesions were the most common manifestation [16]. However, according to reports from one hospital in Korea, pleural lesions were relatively rare (28%), and the frequency of intrapulmonary parenchymal lesions was high (72%) compared to the findings of previous studies [17]. Other differentials, such as aspergillosis, were ruled out because the patient was immunocompetent, and the chest radiograph revealed a cavitated lesion, not compatible with the radiographic pattern associated with this fungi.

A report by Kim et al. has suggested that the main features of chest CT scans of pleuropulmonary paragonimiasis are pulmonary nodules containing a low-attenuation area associated with pleural thickening [16]. Since these clinical manifestations and the radiological appearance of paragonimiasis are similar to those of TB or lung malignancy, the diagnosis is often challenging, leading to improper and inadequate treatment. Other diseases such as aspergillosis or nocardiosis were ruled out as per the immunocompetent status of the patient or the radiological pattern of the disease, the epidemiological data from the region where the patient came from, the clinical progress, and the negative tests and biopsies. 

Other differentials such as pulmonary nocardiosis can involve both the lungs and the central nervous system. The onset may be acute or chronic; symptoms may include fever, fatigue, dyspnea, cough, hemoptysis, and pleuritic chest pain. The isolation of the organism from a clinical specimen establishes the diagnosis. Lung cancer was also considered in the differential. It was then determined that a lung biopsy was needed to confirm the diagnosis.

Treatment of paragonimiasis consists of anthelmintic therapy with praziquantel (75 mg/kg/day in three divided doses for three days), which is generally considered the treatment of choice for all types of paragonimiasis [18]. Triclabendazole (10 mg/kg orally once or twice) is an acceptable first-line agent for treating paragonimiasis in areas where it is available [19]. Our patient received triclabendazole (1250 mg unique dose) as treatment because praziquantel was unavailable in our hospital. The patient responded well to the medication and showed clinical and radiological improvement.

## Conclusions

Paragonimiasis is a parasitic disease caused by the ingestion of raw freshwater crab or crayfish infected with the metacercaria. Given that the clinical manifestations and the radiological appearance of paragonimiasis are similar to those of TB or lung malignancy, the diagnosis is often muddled, leading to improper and inadequate initial treatment. Even though TB should be suspected in areas of high prevalence, other cavitary lung diseases like paragonimiasis should also be part of the differential diagnosis when the AFB in sputum is negative and the patients have dietary habits that make them vulnerable to such diseases.
